# Crystal structure and Hirshfeld surface analysis of a zinc xanthate complex containing the 2,2′-bi­pyridine ligand

**DOI:** 10.1107/S2056989019014968

**Published:** 2019-11-12

**Authors:** Adnan M. Qadir, Sevgi Kansiz, Georgina M. Rosair, Necmi Dege, Inna S. Safyanova

**Affiliations:** a Department of Chemistry, College of Science, Salahaddin University, Erbil, Iraq; b Department of Physics, Faculty of Arts and Sciences, Ondokuz Mayıs University, 55139, Kurupelit, Samsun, Turkey; c Institute of Chemical Sciences, School of Engineering & Physical Sciences, Heriot-Watt University, Edinburgh, EH14 4AS, UK; d Department of Chemistry, Taras Shevchenko National University of Kyiv, 64, Vladimirska Str., Kiev 01601, Ukraine

**Keywords:** crystal structure, xanthate, zinc(II), 2,2′-bi­pyridine, Hirshfeld surface

## Abstract

In the title compound, (2,2′-bi­pyridine-κ^2^ *N*,*N*′)bis­(2-meth­oxy­ethyl xanthato-κ*S*)zinc(II), [Zn(C_4_H_7_O_2_S_2_)_2_(C_10_H_8_N_2_)], the Zn^II^ ion is coordinated to two N atoms of the 2,2′-bi­pyridine ligand and two S atoms from two 2-meth­oxy­ethyl xanthate ligands. The Zn^II^ ion lies on a crystallographic twofold rotation axis and has distorted tetra­hedral coordination geometry. In the crystal, mol­ecules are linked by weak C—H⋯O hydrogen bonds, forming supramolecular chains propagating along the *a*-axis direction. Weak intra­molecular C—H⋯S hydrogen bonds are also observed. The inter­molecular contacts in the crystal were further analysed using Hirshfield surface analysis, which indicates that the most significant contacts are H⋯H (36.3%), followed by S⋯H/H⋯S (24.7%), C⋯H/H⋯C (15.1%), O⋯H/H⋯O (14.4%), N⋯H/H⋯N (4.1%) and C⋯C (2.9%).

## Abstract 2

The Zn^II^ ion lies on a crystallographic twofold axis and has distorted tetra­hedral coordination geometry. Two weak C—H⋯S intra­molecular hydrogen bonds exist between the bipyridyl and thiol groups. In the crystal, mol­ecules are linked by weak C—H⋯O and C—H⋯S hydrogen bonds, forming a three-dimensional supra­molecular architecture.

## Chemical context   

Xanthates (di­thio­carbonates, *R*OCS_2_^−^) have attracted the attention of scientific groups of researchers due to their diverse applications. Metal xanthates have been used as single-source precursors to metal sulfide materials (Kociok-Köhn *et al.*, 2015 ▸). It was reported that metal xanthates have cytotoxic activity on human cancer cells (Efrima *et al.*, 2003 ▸; Friebolin *et al.*, 2005 ▸). Cellulose xanthate have been used for the column separation of alcohols by chromatographic methods (Friebolin *et al.*, 2004 ▸). Zinc(II) xanthate complexes have a tetra­hedral geometry, while zinc(II) xanthate complexes with neutral bidentate nitro­gen donor ligands are either strongly distorted octa­hedral or tetra­hedral. In our previous work, Zn^II^ 2-meth­oxy­ethylxanthate with *N,N,N′,N′*-tetra­methyl­ethyl­ene­di­amine was synthesized, structurally characterized and studied by density functional theory (Qadir *et al.*, 2019 ▸). The complex showed a tetra­hedral environment around metal center and the HOMO–LUMO band gap was 3.9 eV. Aromatic heterocyclic nitro­gen donor ligands have been used by researchers to prepare mixed-ligand complexes of transition metals with supra­moleculer architectures. In this work, the synthesis and crystal structure of a zinc(II) 2-meth­oxy­ethyl xanthate involving 2,2′-bi­pyridine is reported. Hirshfeld surface analysis was used to further investigate the inter­molecular inter­actions.
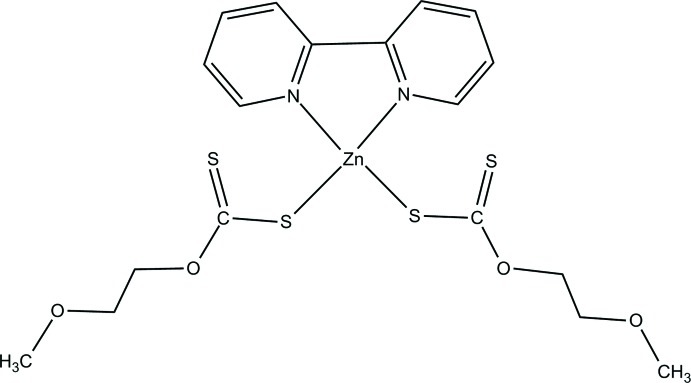


## Structural commentary   

**Figure 1:**
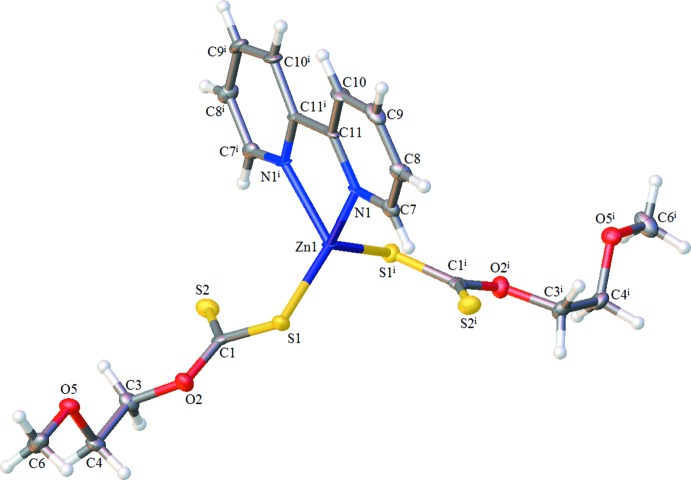
The mol­ecular structure of the title complex, with the atom labelling. Displacement ellipsoids are drawn at the 50% probability level. Symmetry code: (i) 1 − *x*, *y*, 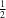 − *z*.

The title complex (Fig. 1 ▸) comprises one Zn^II^ ion, one 2,2′-bi­pyridine ligand and two 2-meth­oxy­ethyl xanthate ligands. The Zn^II^ ion is coordinated to two N atoms of the 2,2′-bi­pyridine ligand and two S atoms from two 2-meth­oxy­ethyl xanthate ligands in a distorted tetra­hedral environment and lies on a crystallographic twofold rotation axis. The Zn—N and Zn—S bond lengths are 2.083 (5) and 2.295 (2) Å, respectively, whereas the bond angles around the central Zn^II^ ion are in the range 78.7 (3)–126.64 (10)° (Table 1 ▸). The bond lengths and angles of the ZnN_2_S_2_ coordination units correspond to those in the structures of mixed-ligand Zn^II^ coordination compounds (see; *Database Survey*). The C—O bond lengths range from 1.346 (8) to 1.453 (8) Å although all of the C—O bonds show single-bond character. In the {S_2_C} part of the xanthate ligands, the C1—S1 distance is 1.727 (7) Å, which is typical of a single bond whereas the C1—S2 distance of 1.652 (7) Å is typical of a carbon-to-sulfur double bond. The C—N and C—C bond lengths in 2,2′-bi­pyridine are normal for 2-substituted pyridine derivatives (Strotmeyer *et al.*, 2003 ▸; Iskenderov *et al.*, 2009 ▸; Golenya *et al.*, 2012 ▸).

## Supra­molecular features   

**Figure 2:**
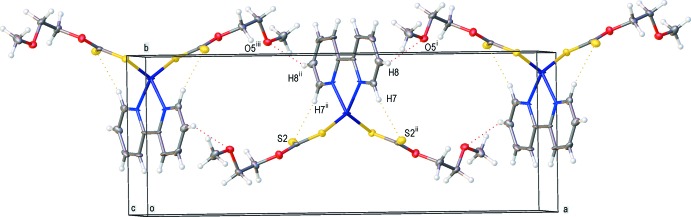
A view of the crystal packing of the title complex. Dashed lines denote the inter­molecular hydrogen bonds (Table 2 ▸). Symmetry codes: (i) 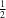 + *x*, 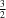 − *y*, 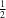 + *z*, (ii) 1 − *x*, *y*, 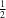 − *z*, (iii) 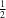 − *x*, 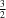 − *y*, −*z.*

The crystal packing of the title compound (Fig. 2 ▸) features inter­molecular C8—H8⋯O5^ii^ hydrogen bonds (Table 2 ▸), which connect the mol­ecules into supramolecular chains propagating along the *a*-axis direction. Weak intra­molecular C—H⋯S hydrogen bonds are also observed.

## Hirshfeld surface analysis   

**Figure 3:**
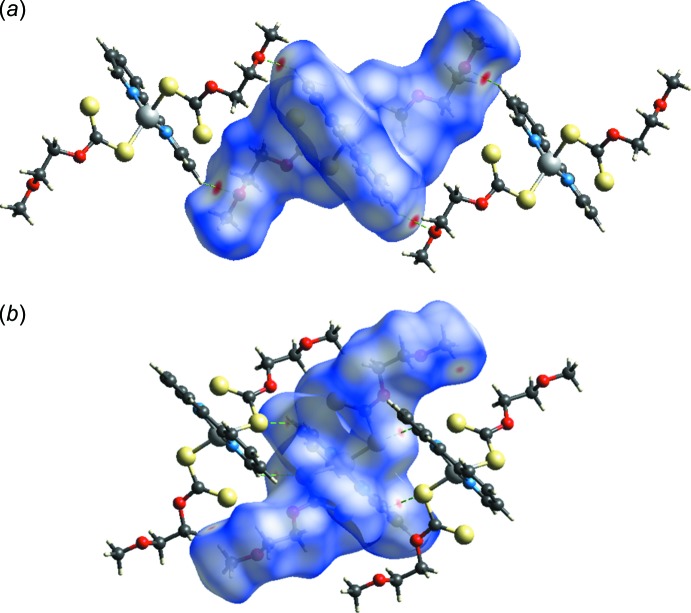
The Hirshfeld surfaces mapped over *d*_norm_ in the range −0.1353 to +1.0127 (arbitrary units) for visualizing the weak inter­molecular (*a*) C—H⋯O and (*b*) C—H⋯S hydrogen bonding.

**Figure 4:**
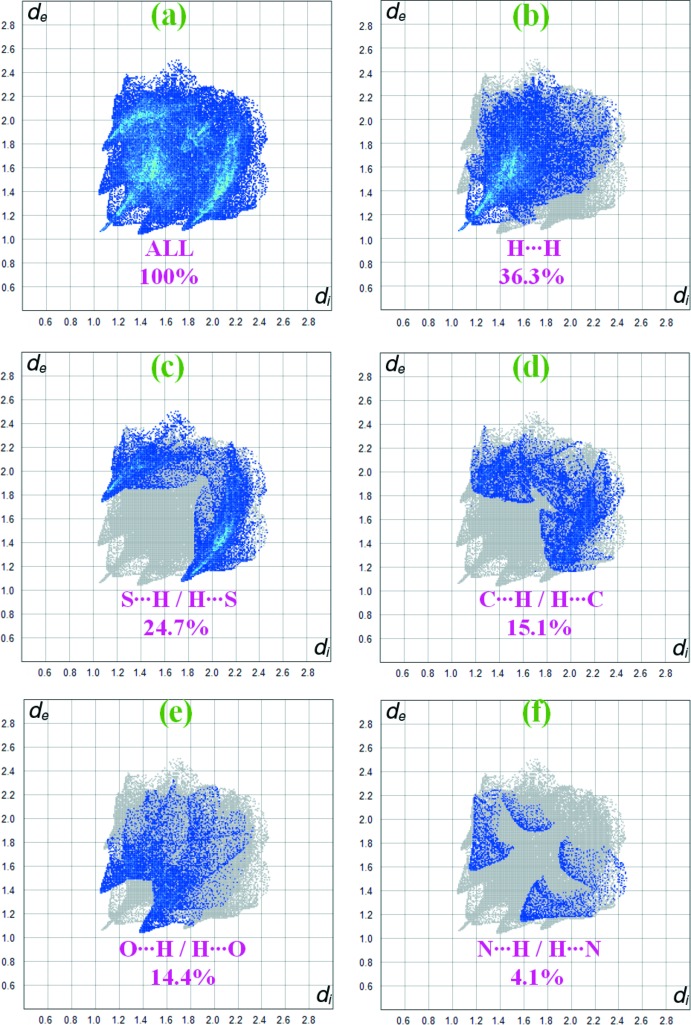
Hirshfeld surface fingerprint plots for the H⋯H, S⋯H/H⋯S, C⋯H/H⋯C and N⋯H/H⋯N contacts of the title complex.

The Hirshfeld surface analysis and the associated two-dimensional fingerprint plots were performed with *CrystalExplorer17.5* (Turner *et al.*, 2017 ▸). The Hirshfeld surface of the title complex is shown in Fig. 3 ▸ *a* and 3*b*. The inter­molecular inter­actions are represented using different colours, red indicating distances closer than the sum of the van der Waals radii, white indicating distances near the van der Waals radii separation, and blue indicating distances longer than the van der Waals radii (McKinnon *et al.*, 2007 ▸). The weak C—H⋯O and C—H⋯S hydrogen bonding in the crystal of the title complex are represented as red spots on *d_norm_*. Selected two-dimensional fingerprint plots are shown in Fig. 4 ▸ for all contacts as well as those delineated into H⋯H, S⋯H/H⋯S and C⋯H/H⋯C contacts, whose percentage contribution is also given. H⋯H inter­molecular contacts make the highest percentage contribution (36.3%), a result of the prevalence of hydrogen from the organic ligands. The S⋯H/H⋯S and O⋯H/H⋯O inter­molecular contacts are due to the attractive C—H⋯S and C—H⋯O hydrogen-bonding inter­actions and make percentage contributions of 24.7 and 14.4%, respectively, indicating these to be the dominant stabilizing inter­actions in this crystal. In addition, C⋯H/H⋯C contacts contribute 15.1% to the Hirshfeld surface. The small percentage contributions from the other different inter­atomic contacts to the Hirshfeld surfaces are as follows: N⋯H/H⋯N (4.1%), C⋯C (2.9%), S⋯S (1.1%), S⋯O/O⋯S (0.8%) and S⋯C/C⋯S (0.3%).

## Database survey   

A search of the Cambridge Structural Database (CSD, version 5.40, update of February 2019; Groom *et al.*, 2016 ▸) for compounds related to the title complex revealed five hits: (2,2′-dipyrid­yl)bis­(butylxanthato)zinc(II) (DIFBOK; Klevtsova *et al.*, 2006 ▸), (2,2′-bi­pyridine)(*O*-*n*-propyl­dithio­carbo­n­ato-κ^2^ *S*,*S*′)(*O*-*n*-propyl­dithio­carbonato-*S*)zinc(II) (IGUGUO; Jeremias *et al.*, 2014 ▸), (2,2′-bi­pyridine)-bis­(*O*-iso­prop­yl­xan­thato)zinc(II) and (2,2′-bi­pyridine)­bis­(*O*-iso­butyl­xan­th­ato)zinc(II) (with refcodes MUJJOQ and MUJJUW, respectively; Klevtsova *et al.*, 2002 ▸) and (2,2′-bipyrid­yl)bis­(ethyl­xan­th­ato)zinc(II) (WITLAM; Glinskaya *et al.*, 2000 ▸). All of these complexes except IGUGUO have tetra­hedral environments around the metal center. The Zn—N and Zn—S bond lengths range from 2.065 to 2.147 Å and 2.284 to 2.341 Å, respectively. The Zn—N and Zn—S bond lengths in the title complex [2.083 (5) and 2.295 (2) Å, respectively] fall within these limits. The structure with refcode IGUGUO has a distorted trigonal–bipyramidal coordination environment.

## Synthesis and crystallization   

To a hot solution of Zn(CH_3_CO_2_). 2H_2_O (10 mmol, 2.20 g) in 2-meth­oxy­ethanol, was added a hot solution of 2,2′-bipy (10 mmol, 1.56 g) in 2-meth­oxy­ethanol. A hot solution of potassium 2-meth­oxy­ethylxanthate (20 mmol, 3.81 g) in 2-meth­oxy­ethanol was added under stirring. Colourless crystals were formed after 30 minutes. The crystals were washed with small amounts of 2-meth­oxy­ethanol and water and air-dried.

## Refinement   

Crystal data, data collection and structure refinement details are summarized in Table 3 ▸. The C-bound H atoms were positioned geometrically and refined using a riding model, with C—H = 0.95, 0.98 and 0.99 Å with *U*_iso_(H) = 1.5*U*_eq_(C) for methyl H atoms and 1.2*U*_eq_(C) otherwise. The crystal was a weak diffractor (*I*/σ at 0.81 resolution was 5.1) and refinedas a two-component twin with HKLF 4 data (twin law −1 0 0 0 − 1 0 0 0 − 1) but this had little effect. The anisotropy of N1 was restrained with ISOR 0.01 0.02 in *SHELXL* (Sheldrick, 2015 ▸).

## Supplementary Material

Crystal structure: contains datablock(s) I. DOI: 10.1107/S2056989019014968/lh5934sup1.cif

Structure factors: contains datablock(s) I. DOI: 10.1107/S2056989019014968/lh5934Isup2.hkl

CCDC references: 1424075, 1424075

Additional supporting information:  crystallographic information; 3D view; checkCIF report

## Figures and Tables

**Table 1:** Selected geometric parameters (Å, °)

Zn1—S1	2.2954 (18)	Zn1—N1	2.083 (5)
			
S1^i^—Zn1—S1	126.64 (10)	N1—Zn1—S1	100.54 (15)
N1^i^—Zn1—S1	120.78 (15)	N1—Zn1—N1^i^	78.7 (3)

Symmetry code: (i) 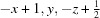.

**Table 2:** Hydrogen-bond geometry (Å, °)

*D*—H⋯*A*	*D*—H	H⋯*A*	*D*⋯*A*	*D*—H⋯*A*
C8—H8⋯O5^i^	0.95	2.51	3.246 (9)	134
C7—H7⋯S2^ii^	0.95	2.90	3.552 (7)	127

Symmetry codes: (i) 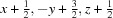; (ii) 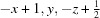.

**Table 3:** Experimental details

Crystal data	
Chemical formula	[Zn(C_4_H_7_O_2_S_2_)_2_(C_10_H_8_N_2_)]
*M*_r_	523.98
Crystal system, space group	Monoclinic, *C*2/*c*
Temperature (K)	100
*a*, *b*, *c* (Å)	22.869 (4), 8.3212 (12), 12.5627 (19)
β (°)	115.348 (4)
*V* (Å^3^)	2160.5 (6)
*Z*	4
Radiation type	Mo *K*α
μ (mm^−1^)	1.55
Crystal size (mm)	0.42 × 0.36 × 0.04

Data collection	
Diffractometer	Bruker APEXII CCD
Absorption correction	Multi-scan (*SADABS*; Bruker, 2009 ▸)
*T*_min_, *T*_max_	0.599, 0.745
No. of measured, independent and observed [*I* > 2σ(*I*)] reflections	11173, 2119, 1954
*R*_int_	0.061
(sin θ/λ)_max_ (Å^−1^)	0.618

Refinement	
*R*[*F* ^2^ > 2σ(*F* ^2^)], *wR*(*F* ^2^), *S*	0.087, 0.155, 1.43
No. of reflections	2119
No. of parameters	134
No. of restraints	6
H-atom treatment	H-atom parameters constrained
Δρ_max_, Δρ_min_ (e Å^−3^)	0.55, −1.00

Computer programs: *APEX2* and *SAINT* (Bruker, 2009 ▸), *SHELXS* (Sheldrick, 2008 ▸), *SHELXL* (Sheldrick, 2015 ▸) and *OLEX2* (Dolomanov *et al.*, 2009 ▸).

## supplementary crystallographic information

### Crystal data

**Table:**

[Zn(C_4_H_7_O_2_S_2_)_2_(C_10_H_8_N_2_)]	*F*(000) = 1080
*M**_r_* = 523.98	*D*_x_ = 1.611 Mg m^−^^3^
Monoclinic, *C*2/*c*	Mo *K*α radiation, λ = 0.71073 Å
*a* = 22.869 (4) Å	Cell parameters from 2844 reflections
*b* = 8.3212 (12) Å	θ = 3.0–25.4°
*c* = 12.5627 (19) Å	µ = 1.55 mm^−^^1^
β = 115.348 (4)°	*T* = 100 K
*V* = 2160.5 (6) Å^3^	Plate, colourless
*Z* = 4	0.42 × 0.36 × 0.04 mm

### Data collection

**Table:**

Bruker APEXII CCD diffractometer	1954 reflections with *I* > 2σ(*I*)
φ and ω scans	*R*_int_ = 0.061
Absorption correction: multi-scan (SADABS; Bruker, 2009)	θ_max_ = 26.1°, θ_min_ = 2.6°
*T*_min_ = 0.599, *T*_max_ = 0.745	*h* = −28→28
11173 measured reflections	*k* = −9→10
2119 independent reflections	*l* = −14→15

### Refinement

**Table:**

Refinement on *F*^2^	Primary atom site location: structure-invariant direct methods
Least-squares matrix: full	Hydrogen site location: inferred from neighbouring sites
*R*[*F*^2^ > 2σ(*F*^2^)] = 0.087	H-atom parameters constrained
*wR*(*F*^2^) = 0.155	*w* = 1/[σ^2^(*F*_o_^2^) + 39.0236*P*] where *P* = (*F*_o_^2^ + 2*F*_c_^2^)/3
*S* = 1.43	(Δ/σ)_max_ < 0.001
2119 reflections	Δρ_max_ = 0.55 e Å^−^^3^
134 parameters	Δρ_min_ = −1.00 e Å^−^^3^
6 restraints	

### Special details

**Table:**

Geometry. All esds (except the esd in the dihedral angle between two l.s. planes) are estimated using the full covariance matrix. The cell esds are taken into account individually in the estimation of esds in distances, angles and torsion angles; correlations between esds in cell parameters are only used when they are defined by crystal symmetry. An approximate (isotropic) treatment of cell esds is used for estimating esds involving l.s. planes.
Refinement. Refined as a 2-component inversion twin.

### Fractional atomic coordinates and isotropic or equivalent isotropic displacement parameters (Å^2^)

**Table:**

	*x*	*y*	*z*	*U*_iso_*/*U*_eq_	
Zn1	0.5000	0.63086 (13)	0.2500	0.0085 (3)	
S1	0.44329 (8)	0.5070 (2)	0.33978 (16)	0.0164 (4)	
S2	0.36008 (9)	0.4541 (2)	0.08028 (17)	0.0208 (4)	
O2	0.3340 (2)	0.3748 (7)	0.2598 (4)	0.0203 (11)	
O5	0.2155 (2)	0.4123 (6)	0.2833 (4)	0.0193 (11)	
N1	0.5396 (3)	0.8245 (6)	0.3634 (5)	0.0101 (11)	
C1	0.3750 (3)	0.4405 (8)	0.2208 (6)	0.0153 (15)	
C3	0.2717 (3)	0.3151 (9)	0.1750 (6)	0.0186 (16)	
H3A	0.2775	0.2208	0.1325	0.022*	
H3B	0.2481	0.3996	0.1169	0.022*	
C4	0.2350 (4)	0.2688 (9)	0.2455 (7)	0.0192 (16)	
H4A	0.1966	0.2038	0.1964	0.023*	
H4B	0.2630	0.2037	0.3146	0.023*	
C6	0.1815 (4)	0.3775 (11)	0.3521 (7)	0.0289 (19)	
H6A	0.1441	0.3094	0.3067	0.043*	
H6B	0.1666	0.4780	0.3731	0.043*	
H6C	0.2102	0.3211	0.4240	0.043*	
C7	0.5764 (3)	0.8146 (9)	0.4798 (6)	0.0165 (15)	
H7	0.5905	0.7116	0.5138	0.020*	
C8	0.5948 (4)	0.9481 (9)	0.5521 (6)	0.0191 (16)	
H8	0.6204	0.9372	0.6345	0.023*	
C9	0.5751 (4)	1.0972 (9)	0.5021 (7)	0.0214 (17)	
H9	0.5874	1.1913	0.5494	0.026*	
C10	0.5371 (3)	1.1084 (8)	0.3815 (7)	0.0189 (16)	
H10	0.5229	1.2103	0.3455	0.023*	
C11	0.5204 (3)	0.9708 (8)	0.3151 (6)	0.0113 (14)	

### Atomic displacement parameters (Å^2^)

**Table:**

	*U*^11^	*U*^22^	*U*^33^	*U*^12^	*U*^13^	*U*^23^
Zn1	0.0137 (6)	0.0030 (5)	0.0118 (6)	0.000	0.0084 (4)	0.000
S1	0.0169 (9)	0.0160 (9)	0.0181 (9)	−0.0021 (7)	0.0091 (7)	0.0031 (7)
S2	0.0285 (10)	0.0198 (10)	0.0198 (10)	−0.0027 (8)	0.0158 (8)	−0.0026 (8)
O2	0.018 (3)	0.022 (3)	0.023 (3)	−0.002 (2)	0.011 (2)	0.003 (2)
O5	0.022 (3)	0.016 (3)	0.023 (3)	−0.001 (2)	0.013 (2)	0.002 (2)
N1	0.016 (3)	0.003 (3)	0.017 (3)	−0.004 (2)	0.011 (2)	−0.004 (2)
C1	0.019 (4)	0.007 (3)	0.022 (4)	0.004 (3)	0.011 (3)	0.004 (3)
C3	0.020 (4)	0.019 (4)	0.017 (4)	−0.006 (3)	0.008 (3)	−0.005 (3)
C4	0.020 (4)	0.014 (4)	0.023 (4)	−0.005 (3)	0.009 (3)	−0.001 (3)
C6	0.028 (4)	0.029 (4)	0.038 (5)	0.009 (4)	0.022 (4)	0.015 (4)
C7	0.017 (4)	0.018 (4)	0.020 (4)	−0.002 (3)	0.013 (3)	−0.003 (3)
C8	0.025 (4)	0.021 (4)	0.016 (4)	−0.006 (3)	0.013 (3)	−0.003 (3)
C9	0.021 (4)	0.018 (4)	0.030 (4)	−0.008 (3)	0.015 (3)	−0.014 (3)
C10	0.020 (4)	0.006 (3)	0.032 (4)	−0.002 (3)	0.013 (3)	−0.005 (3)
C11	0.012 (3)	0.004 (3)	0.021 (4)	−0.002 (3)	0.011 (3)	−0.002 (3)

### Geometric parameters (Å, º)

**Table:**

Zn1—S1	2.2954 (18)	C4—H4A	0.9900
Zn1—S1^i^	2.2954 (18)	C4—H4B	0.9900
Zn1—N1^i^	2.083 (5)	C6—H6A	0.9800
Zn1—N1	2.083 (5)	C6—H6B	0.9800
S1—C1	1.727 (7)	C6—H6C	0.9800
S2—C1	1.652 (7)	C7—H7	0.9500
O2—C1	1.346 (8)	C7—C8	1.382 (10)
O2—C3	1.453 (8)	C8—H8	0.9500
O5—C4	1.426 (9)	C8—C9	1.377 (11)
O5—C6	1.418 (8)	C9—H9	0.9500
N1—C7	1.342 (9)	C9—C10	1.390 (11)
N1—C11	1.347 (8)	C10—H10	0.9500
C3—H3A	0.9900	C10—C11	1.371 (9)
C3—H3B	0.9900	C11—C11^i^	1.497 (13)
C3—C4	1.506 (10)		
			
S1^i^—Zn1—S1	126.64 (10)	C3—C4—H4A	110.0
N1^i^—Zn1—S1	120.78 (15)	C3—C4—H4B	110.0
N1—Zn1—S1^i^	120.78 (15)	H4A—C4—H4B	108.4
N1—Zn1—S1	100.54 (15)	O5—C6—H6A	109.5
N1^i^—Zn1—S1^i^	100.54 (15)	O5—C6—H6B	109.5
N1—Zn1—N1^i^	78.7 (3)	O5—C6—H6C	109.5
C1—S1—Zn1	102.2 (2)	H6A—C6—H6B	109.5
C1—O2—C3	119.3 (5)	H6A—C6—H6C	109.5
C6—O5—C4	111.4 (6)	H6B—C6—H6C	109.5
C7—N1—Zn1	125.7 (5)	N1—C7—H7	118.7
C7—N1—C11	118.5 (6)	N1—C7—C8	122.7 (7)
C11—N1—Zn1	115.4 (4)	C8—C7—H7	118.7
S2—C1—S1	126.8 (4)	C7—C8—H8	120.8
O2—C1—S1	109.1 (5)	C9—C8—C7	118.4 (7)
O2—C1—S2	124.0 (5)	C9—C8—H8	120.8
O2—C3—H3A	110.5	C8—C9—H9	120.4
O2—C3—H3B	110.5	C8—C9—C10	119.2 (7)
O2—C3—C4	105.9 (6)	C10—C9—H9	120.4
H3A—C3—H3B	108.7	C9—C10—H10	120.4
C4—C3—H3A	110.5	C11—C10—C9	119.2 (7)
C4—C3—H3B	110.5	C11—C10—H10	120.4
O5—C4—C3	108.3 (6)	N1—C11—C10	121.9 (6)
O5—C4—H4A	110.0	N1—C11—C11^i^	115.0 (4)
O5—C4—H4B	110.0	C10—C11—C11^i^	123.1 (4)
			
Zn1—S1—C1—S2	4.2 (5)	C3—O2—C1—S2	−1.8 (9)
Zn1—S1—C1—O2	−175.3 (4)	C6—O5—C4—C3	−179.0 (6)
Zn1—N1—C7—C8	−172.1 (5)	C7—N1—C11—C10	−0.5 (9)
Zn1—N1—C11—C10	173.2 (5)	C7—N1—C11—C11^i^	179.9 (6)
Zn1—N1—C11—C11^i^	−6.4 (9)	C7—C8—C9—C10	0.7 (10)
O2—C3—C4—O5	73.0 (7)	C8—C9—C10—C11	−0.3 (10)
N1—C7—C8—C9	−0.9 (10)	C9—C10—C11—N1	0.2 (10)
C1—O2—C3—C4	−173.4 (6)	C9—C10—C11—C11^i^	179.8 (7)
C3—O2—C1—S1	177.8 (5)	C11—N1—C7—C8	0.8 (10)

Symmetry code: (i) −*x*+1, *y*, −*z*+1/2.

### Hydrogen-bond geometry (Å, º)

**Table:**

*D*—H···*A*	*D*—H	H···*A*	*D*···*A*	*D*—H···*A*
C8—H8···O5^ii^	0.95	2.51	3.246 (9)	134
C7—H7···S2^i^	0.95	2.90	3.552 (7)	127

Symmetry codes: (i) −*x*+1, *y*, −*z*+1/2; (ii) *x*+1/2, −*y*+3/2, *z*+1/2.
